# Management of 10 or More Uterine Fibroids by Laparoscopic Myomectomy: A Comprehensive Review

**DOI:** 10.7759/cureus.92553

**Published:** 2025-09-17

**Authors:** Shweta More, Kunal Rathod

**Affiliations:** 1 Obstetrics and Gynaecology, Barking, Havering and Redbridge University Hospitals NHS Trust, London, GBR

**Keywords:** adhesion barrier, fertility, fibroids, haemostasis, laparoscopic myomectomy

## Abstract

Laparoscopic myomectomy (LM) for 10 or more fibroids combines the fertility-preserving benefits of minimally invasive surgery with the technical challenge of haemostasis and uterine reconstruction. This narrative review draws exclusively on randomized controlled trials (RCTs), systematic reviews, and meta-analyses to outline perioperative bleeding-reduction strategies, advanced surgical techniques, postoperative complications, and reproductive outcomes. This review highlights structured perioperative pathways with the integration of medical pretreatment, standardized intraoperative protocols, and postoperative care bundle with impact on resource utilization as well as overall patient outcomes. Multimodal preoperative optimization of individual patients involving pharmacotherapy, various collaborative intraoperative haemostasis techniques, precise stepwise surgical approach during LM, and vigilant postoperative management to identify as well as manage the postoperative complications improves overall patient safety. This review article focuses on detailed preoperative counselling to match realistic expectations of desirable fertility outcomes. The quality-of-life assessments and patient-reported outcome measures should be integrated into future research to capture the broader impact on reproductive health in these patients.

## Introduction and background

Uterine leiomyomas affect up to 70% of women by age 50, and nearly 15% present with multiple lesions exceeding 10 in number [1]. Compared with open myomectomy, laparoscopic myomectomy (LM) offers less pain, shorter hospitalization, and faster recovery. However, large series confirm that excising more than 10 uterine fibroids carries increased operative time, increased bleeding risks, bloodless enucleation of fibroids, increased average duration of hospital stay, and higher conversion rate to laparotomy [2]. Meta-analyses demonstrate that, in experienced hands, LM reduces blood loss by 150 mL on average versus laparotomy while maintaining similar complication rates [3]. LM has a distinct advantage of being a fertility-preserving option along with the aim of developing preoperative haemostatic techniques and advanced surgical techniques to reduce the risk of postoperative complications. This review article aims to provide a narrative summary of the recent evidence in relation to outlining perioperative bleeding-reduction strategies, advanced surgical techniques, postoperative complications, and reproductive outcomes in this subgroup of patients undergoing LM. 

## Review

Preoperative bleeding-reduction strategies

Gonadotropin-releasing hormone (GnRH) agonists have become a cornerstone of preoperative preparation for high-burden fibroid cases. By downregulating the pituitary release of luteinizing hormone (LH) and follicle-stimulating hormone (FSH), agents such as leuprolide acetate or goserelin induce a hypoestrogenic state that shrinks fibroid volume by 30-50% over an 8-12-week course, softens the pseudocapsule, and corrects anaemia through improved endometrial haemostasis [4]. In the landmark randomized controlled trial (RCT) of 180 women randomized to three months of leuprolide versus placebo, those receiving GnRH agonist experienced a mean blood loss of 115 mL compared with 210 mL in controls, a 45% reduction alongside a significant drop in intraoperative transfusion rates (5% vs. 18%; p<0.01), and easier enucleation due to reduced tissue turgor [4]. Drawbacks include menopausal symptoms (hot flushes in 80%), decreased bone mineral density with courses beyond six months, potential rebound fibroid growth if surgery is delayed, and a theoretical increase in operative difficulty once drug effects wane [4].

Selective progesterone receptor modulators (SPRMs), most notably ulipristal acetate, offer an alternative by directly antagonizing progesterone's proliferative effect on fibroid cells. In a multicentre RCT of 102 women pretreated for 12 weeks, ulipristal matched leuprolide's fibroid volume reduction (35-45%) but with a markedly lower incidence of hypoestrogenic symptoms (12% vs. 58%; p<0.001) and rapid control of heavy menstrual bleeding [5]. Treatment is typically limited to three courses of three months each, given rare reports of liver enzyme elevations, necessitating periodic transaminase monitoring. Post-treatment rebound remains a concern, though some series suggest more sustained shrinkage than GnRH agonists.

Antifibrinolytic therapy with tranexamic acid (TXA) stabilizes clot formation at the surgical field by blocking plasminogen activation. A meta-analysis of six RCTs encompassing 620 patients demonstrated that a single 1 g IV bolus of TXA administered immediately before incision reduced mean intraoperative blood loss by 115 mL (28%) without an associated rise in venous thromboembolism (VTE) or ischaemic events. Further benefit was seen when TXA was continued as a postoperative infusion for 12 hours, although concerns persist in patients with prior VTE, and caution is advised in those with severe renal impairment [6].

Preoperative uterine artery embolization (PUAE) employs interventional radiology to transiently occlude the uterine arterial supply with particles or coils 24-48 hours before LM. A systematic review of cohort studies revealed consistent reductions in estimated blood loss (EBL) exceeding 50%, lower intraoperative transfusion requirements, and smaller fibroid volume at enucleation. However, PUAE carries its own risks: post-embolization syndrome (pelvic pain, low-grade fever, nausea) in up to 30% of patients, potential compromise of ovarian reserve if collaterals are embolized, and the logistical need for interventional radiology availability [7].

Finally, optimizing haematologic status remains essential. Oral iron supplementation (e.g., ferrous sulphate 325 mg three times daily) corrects mild anaemia over 4-8 weeks but often causes gastrointestinal upset. Intravenous iron formulations (iron sucrose or ferric carboxymaltose) replenish stores more rapidly, raising haemoglobin by 1-2 g/dL within 2-3 weeks [8]. In patients with severe anaemia (Hb <10 g/dL), adjunctive erythropoietin alpha (40,000 U weekly) has been shown in cohort series to halve transfusion rates, though its use is tempered by cost and a small thrombotic risk [8]. Altogether, a multimodal preoperative regimen, combining medical fibroid shrinkage, antifibrinolytics, arterial devascularization, and anaemia correction, sets the stage for safer laparoscopic removal of more than 10 fibroids (Table 1).

**Table 1:** Overview of the preoperative bleeding-reduction strategies GnRH: gonadotropin-releasing hormone; LH: luteinizing hormone; FSH: follicle-stimulating hormone; RCT: randomized controlled trial; EBL: estimated blood loss; SPRM: selective progesterone receptor modulator; TXA: tranexamic acid; VTE: venous thromboembolism; PUAE: preoperative uterine artery embolization; IR: interventional radiology; EPO: erythropoietin; GI: gastrointestinal

Intervention	Mechanism	Evidence and outcomes	Pros	Cons
GnRH agonists (leuprolide, goserelin) [4]	Downregulate LH/FSH → hypoestrogenism; fibroid shrinkage	RCT (n=180): 8-12 weeks of leuprolide → 30-50% fibroid volume reduction, mean EBL 115 mL vs. 210 mL (-45%), transfusion 5% vs. 18% (p<0.01)	Significant shrinkage eases enucleation and reduces blood loss and transfusions	Hot flushes in 80%, bone density loss if >6 months, rebound growth if surgery delayed
SPRM (ulipristal acetate) [5]	Progesterone receptor blockade → inhibits fibroid growth	Multicentre RCT (n=102): 12 weeks of ulipristal → 35-45% volume reduction, hypoestrogenic symptoms 12% vs. 58% (p<0.001)	Comparable shrinkage to GnRH agonists, fewer menopausal symptoms, rapid bleeding control	Limited to three 3-month courses, requires liver function monitoring, potential rebound
TXA [6]	Antifibrinolytic → stabilizes clot formation	Meta-analysis of 6 RCTs (n=620): single 1 g IV bolus → mean EBL reduction 115 mL (-28%), no ↑ VTE or ischaemic events	Simple dosing, well tolerated, effective 28% blood loss reduction	Caution in patients with prior VTE, use with severe renal impairment
PUAE [7]	IR occlusion of uterine arteries 24-48 hours pre-surgery	Systematic review of cohorts: >50% reduction in EBL; lower transfusion rates; smaller fibroid volume at enucleation	Dramatic blood loss reduction, may convert open to laparoscopic approach	Post-embolization syndrome in ~30% (pain, fever), risk to ovarian reserve, needs IR suite
Anaemia optimization (iron ± erythropoietin) [8]	↑ haemoglobin reserve via oral/IV iron or EPO	Cohort series: IV iron ↑ haemoglobin by 1-2 g/dL in 2-3 weeks; EPO α (40,000 U weekly) halved transfusion rates	Builds margin against intra-op bleeding, supports healing	GI upset with oral iron, cost and thrombotic risk with EPO

Intraoperative haemostatic techniques

**Figure 1:**
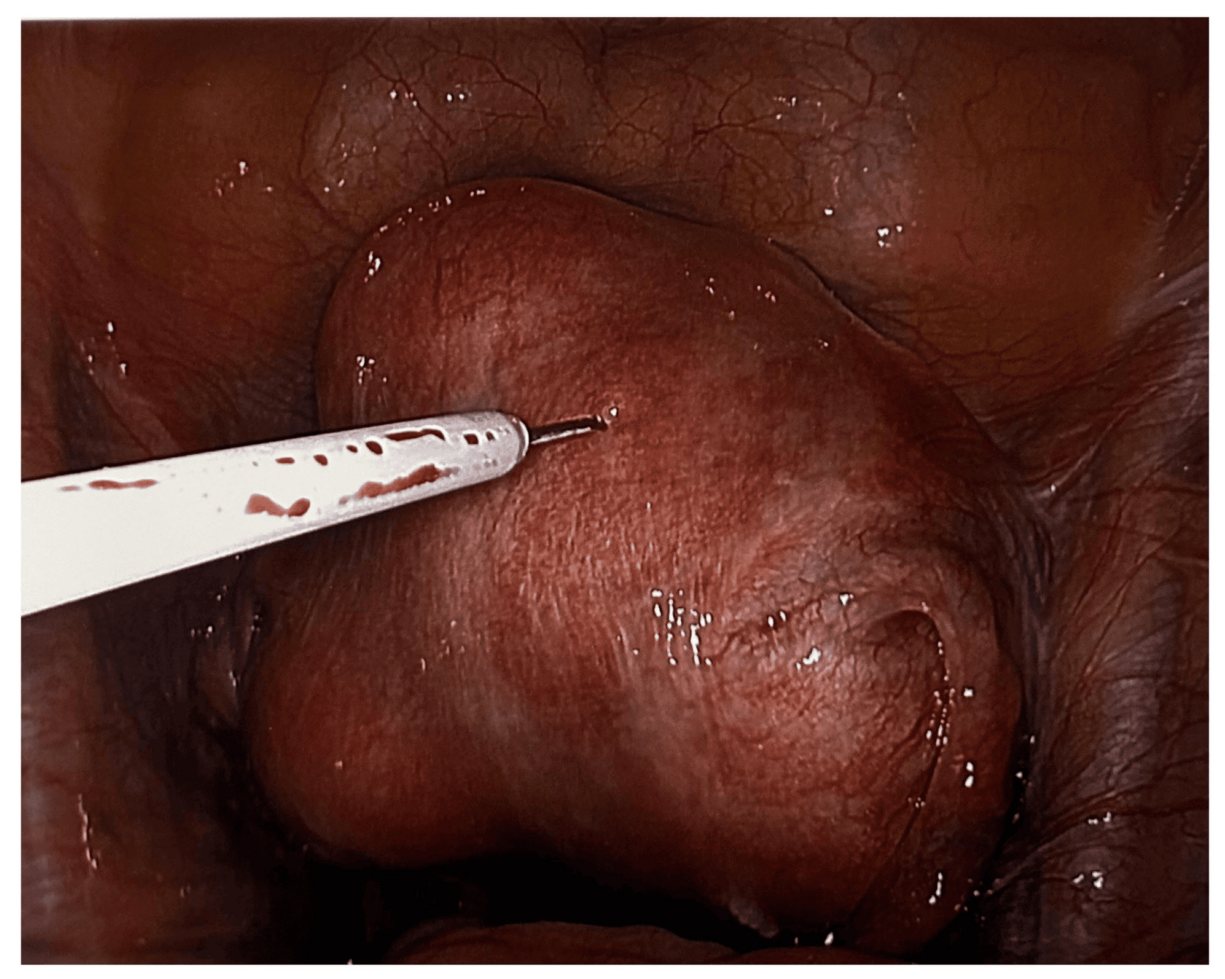
Intramyometrial vasopressin injection: intraoperative haemostatic technique

The myometrial vasopressin injection acts on V1 receptors in the uterine microvasculature to induce potent, short-lived vasoconstriction. In the pivotal RCT of 150 women undergoing LM, surgeons injected 0.05 U/mL vasopressin, total dose capped at 4 U in 1 mL aliquots, directly into the myometrium at 3-4 o'clock positions around each fibroid. The blanching effect was visible within 30 seconds and sustained for 20-30 minutes, covering multiple enucleations without re-dosing. Mean EBL fell from 210 mL to 115 mL (p<0.001), transfusion rates dropped from 12% to 4%, and procedural clarity improved, reducing reliance on bipolar cautery [9]. Cardiovascular monitoring revealed no serious sequelae, though transient bradycardia occurred in 2% of cases [9]. Key pitfalls include inadvertent intravascular injection, avoided by aspirating before each bolus, and strict dose limits to prevent systemic vasoconstriction (Figure 1).

The silicone uterine tourniquet is a simple, reversible device that compresses uterine arteries at the lower segment. In a single-centre RCT (n=130), investigators fashioned a loop of 7 mm silicone vessel loop around the isthmus and tightened it just before the first capsular incision; removal occurred immediately after all fibroids were enucleated [10]. This achieved a 35% reduction in EBL without pharmacologic side effects, shortening the need for extensive energy use and expediting suturing. Tourniquet placement added under two minutes to setup time, but surgeons noted that over-tightening or prolonged occlusion (>30 minutes) risked focal ischaemia of the lower uterine segment. Careful release intervals after every two to three fibroids can mitigate this [10].

Temporary laparoscopic uterine artery occlusion, which is based on open surgical techniques, involves the laparoscopic clipping of the ascending uterine arteries and leads to precise regional devascularization. A meta-analysis of three RCTs (total n=280) compared bipolar clipping of each uterine artery at its origin on the internal iliac versus standard LM [11]. Pooled results showed a 38% decrease in mean EBL (MD -135 mL; p<0.001) and a drop in transfusions from 9% to 3%, with no increase in ureteral injury. Surgeons performed retroperitoneal dissection to visualize the uterine artery in <10 minutes per side. Challenges include the learning curve for safe retroperitoneal access and extended operative time by roughly 15 minutes, although this was offset by reduced haemostasis time [11].

Energy-based vessel sealing devices, like advanced bipolar (Liga Sure, Medtronic, Dublin, Ireland) and ultrasonic (Harmonic ACE, Ethicon, Raritan, New Jersey, United States) platforms, seal vessels up to 7 mm via thermal or mechanical compression.** **Five RCTs (n=420) comparing these devices in LM found no significant difference in EBL, but ultrasonic shears shortened overall operative time by an average of 12 minutes (p=0.02) [12]. Surgeons appreciated the Harmonic's ability to dissect along tissue planes with minimal lateral thermal spread, reducing collateral myometrial damage. Bipolar energy offered higher burst pressures, which some preferred for thick fibroid pseudocapsules. The choice often reflects surgeon comfort, device availability, and cost considerations [12].

Barbed sutures for myometrial closure (e.g., V-Loc, Medtronic, Dublin, Ireland) anchor automatically, eliminating intracorporeal knot-tying. In four RCTs (n=610), barbed sutures reduced uterine closure time by seven minutes (p<0.001) and EBL by 20% versus conventional Vicryl [13]. Even tension distribution along the entire suture line minimized bleeding from needle tracks. On magnetic resonance imaging (MRI) at six months, uterine scars were comparable. Surgeons must ensure secure anchoring at the suture's start and avoid entanglement, but once mastered, this technique streamlines multilayer closure in high-burden LM [13].

Topical haemostatic agents, like fibrin sealants, gelatin-thrombin matrices, and oxidized regenerated cellulose, can be applied directly to raw myometrial beds in situations with persistent oozing despite pharmacologic and mechanical measures. A systematic review (n>200) confirmed that these adjuncts reduced minor bleeds and transfusion rates but cautioned against overuse due to elevated cost and a small risk of adhesion formation [14]. The best practice is to limit the application to focal bleeding areas and remove excess after achieving haemostasis.

Controlled hypotensive anaesthesia by lowering mean arterial pressure (MAP) by 20-30 mmHg via titrated anaesthetics or short‐acting vasodilators uniformly diminishes uterine perfusion. An RCT of 120 American Society of Anesthesiologists (ASA) grade I-II women demonstrated a 25% reduction in EBL with MAP maintained at 60-65 mmHg, without adverse renal or neurologic effects [15]. This approach synergizes with local haemostatic methods, though it requires invasive monitoring and is contraindicated in those with significant cardiovascular or cerebrovascular disease (Table 2).

**Table 2:** Overview of the intraoperative haemostatic techniques RCT: randomized controlled trial; EBL: estimated blood loss

Technique	Evidence (study, n)	Efficacy	Pros	Cons
Intramyometrial vasopressin injection [9]	RCT (n=150)	EBL ↓ from 210 mL to 115 mL (-45%); transfusion from 12% to 4%	Rapid, field-wide blanching; covers multiple enucleations	Transient bradycardia (2%); risk if injected intravascularly
Silicone uterine tourniquet [10]	RCT (n=130)	EBL ↓ 35%	Reversible mechanical devascularization; no drugs required	Obscures lower segment view; focal ischaemia if >30 min
Temporary uterine artery occlusion [11]	Meta-analysis of RCTs (n=280)	EBL ↓ 38% (MD –135 mL); transfusion from 9% to 3%	Precise regional control; removable before closure	Requires retroperitoneal dissection; adds ~15 min
Energy-based vessel sealing devices [12]	Meta-analysis of RCTs (n=420)	EBL: same; operative time ↓ 12 min	Ultrasonic: fine dissection, minimal lateral spread	Higher equipment cost; device availability varies
Barbed sutures for myometrial closure [13]	Meta-analysis of RCTs (n=610)	Closure time ↓ 7 min; EBL ↓ 20%	Knotless; uniform tension distribution; faster closure	Learning curve; risk of suture entanglement
Topical haemostatic agents [14]	Systematic review (n>200)	Reduces minor oozing and transfusion needs	Adjunct for diffuse bleeding; biodegradable	High cost; potential adhesion formation
Controlled hypotensive anaesthesia [15]	RCT (n=120)	EBL ↓ ~25%	Synergistic with local measures; non-surgical	Requires invasive monitoring; contraindicated in cardiovascular disease

Advanced surgical techniques

**Figure 2:**
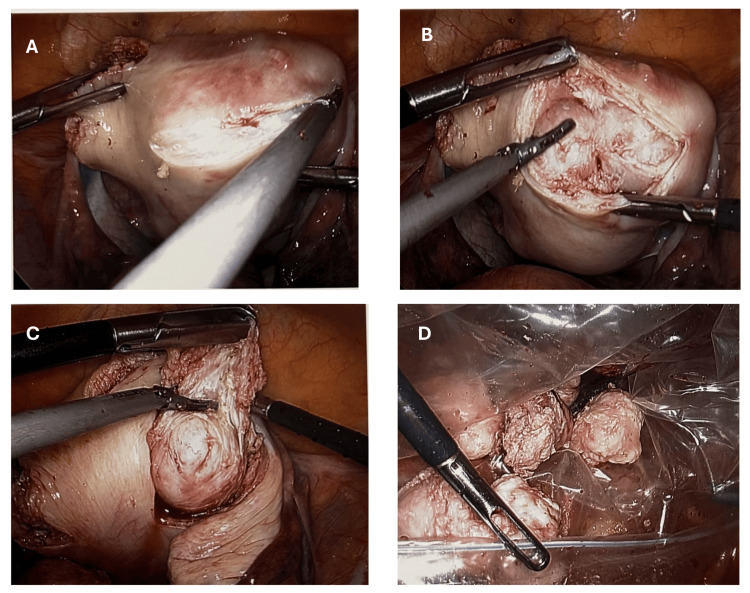
Advanced surgical techniques for myomectomy: (A) transverse serosa-parallel uterine incision, (B) capsular dissection by identifying the plane between fibroids and pseudocapsule, (C) enucleation of fibroid, and (D) enclosed fibroids in an endobag

Transverse serosal-parallel uterine incisions capitalize on the natural vascular anatomy of the myometrium. By aligning the incision with the subserosal plexus, surgeons preserve collateral blood flow and reduce devascularized tissue margins (Figure 2A). In the randomized trial of 160 women, transverse incisions yielded a 15% reduction in EBL compared to vertical incisions (mean EBL 130 mL vs. 153 mL; p=0.02), likely due to the less interruption of arcuate arteries and more efficient closure of the muscular edges [16]. Practically, the incision is made 1 cm over the fibroid apex, extending transversely just wide enough to expose the pseudocapsule without unnecessary myometrial sacrifice.

Sharp capsular dissection using cold scissors further limits thermal injury and preserves healthy myometrium (Figure 2B). Rather than relying on energy devices, surgeons dissect along the natural cleavage plane between the fibroid pseudocapsule and surrounding tissue, gently teasing the leiomyoma free. Narrative reviews highlight that this technique maintains tissue integrity, reduces collateral damage, and facilitates precise tactile feedback, critical when multiple fibroids require sequential enucleation (Figure 2C). The result is less charred tissue edges, which improves the ability to suture and may decrease postoperative adhesion formation. In-bag contained power morcellation addresses concerns about tissue dissemination and parasitic myoma development [17]. In a landmark RCT of 200 patients, use of a specialized containment bag eliminated the intraperitoneal spread of fibroid fragments, whereas uncontained morcellation left parasitic myomas in 8% of cases at six-month follow-up laparoscopies (p<0.001) [18]. The bag is deployed before myoma extraction, insufflated to create working space, and then carefully retrieved, adding only 10-15 minutes to operative time while safeguarding against occult sarcoma dissemination and peritoneal seeding (Figure 2D).

Double-layer myometrial closure restores uterine wall strength and minimizes adhesion risks. A meta-analysis of 14 RCTs demonstrated that a two-layer closure, first approximating deep myometrial edges with interrupted or continuous absorbable sutures, followed by a seromuscular imbricating layer, halved postoperative adhesions compared with single-layer techniques (adhesion rates 18% vs. 36%; p=0.01) [19]. This method also uniformizes tension across the repair, reducing niche formation and supporting optimal healing for future pregnancies.

**Figure 3:**
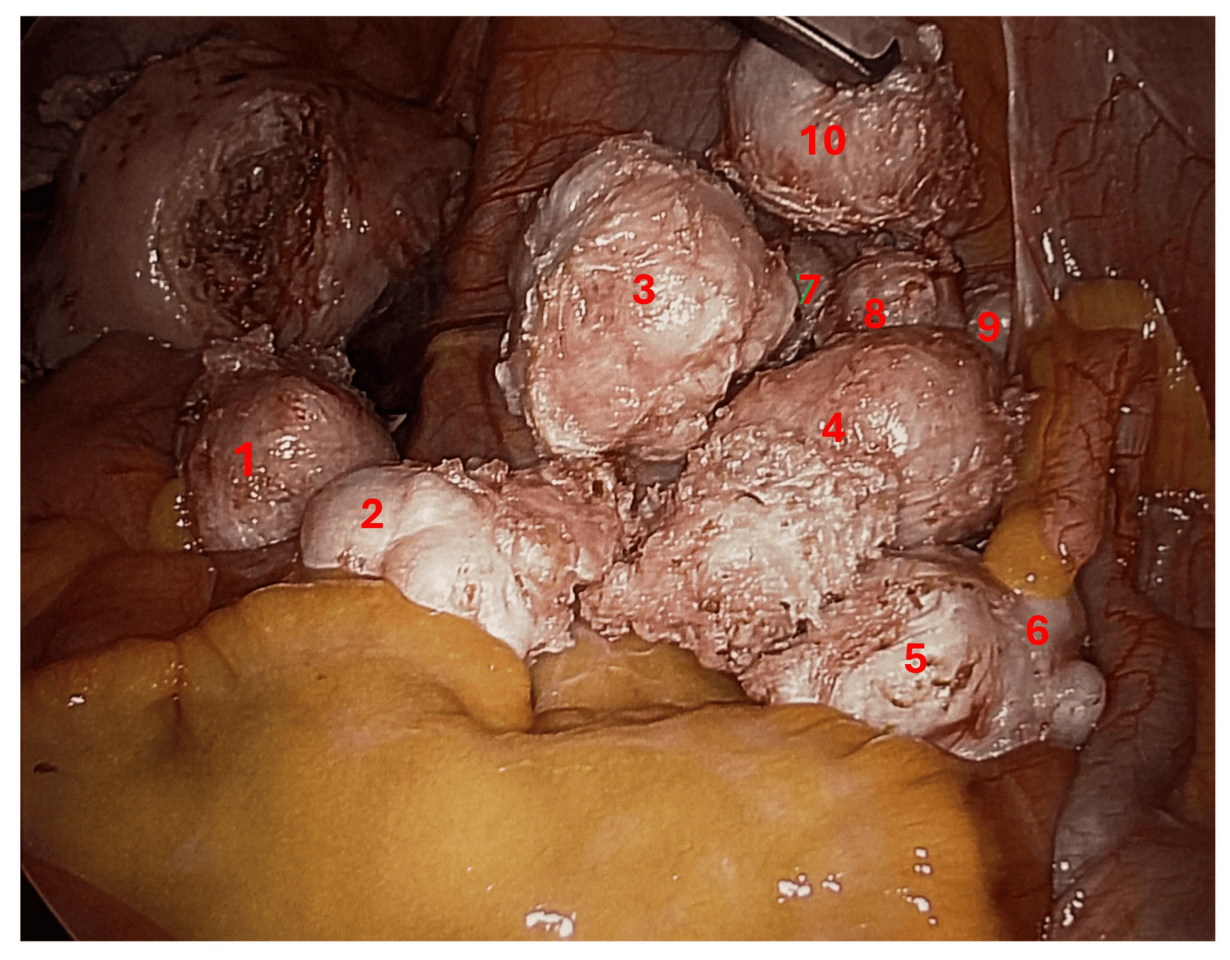
Intraoperative view of enucleated multiple fibroids (10 in this patient as numbered)

Systematic reviews comparing laparoscopic and open myomectomy in women with high fibroid burden confirm the minimally invasive approach's advantages. Pooled data show that LM reduces mean blood loss by roughly 150 mL, shortens hospital stay by 2.5 days, and maintains a low conversion rate to laparotomy of 3%, without increasing overall complication rates [20]. Although operative time is 30-45 minutes longer, patients benefit from less postoperative pain, earlier return to activity, and superior cosmetic outcomes (Figure 3).

Robotic-assisted myomectomy offers enhanced dexterity for complex suturing and multi-fibroid enucleation. Retrospective series indicate similar haemostatic results to conventional laparoscopy but with fewer conversions in extremely fibroid-dense uteri, attributed to the wristed instruments and three-dimensional visualization [21]. However, its higher cost and longer docking times remain barriers, and comparative RCTs are needed to define its precise role when tackling more than 10 fibroids (Table 3).

**Table 3:** Overview of advanced surgical techniques RCT: randomized controlled trial; EBL: estimated blood loss

Technique	Description and mechanism	Evidence and outcomes	Advantages	Limitations
Transverse serosal-parallel incision [16]	Incision aligned with subserosal vascular plexus to preserve collateral flow	RCT (n=160): mean EBL 130 mL vs. 153 mL (-15%; p=0.02)	Less bleeding; efficient muscular edge closure	Requires precise placement; too small incision limits access
Sharp capsular dissection with cold scissors [17]	Sharp dissection along pseudocapsule preserves myometrium by avoiding thermal injury	Narrative reviews: improved tissue integrity, reduced collateral damage	Better tactile feedback; may reduce adhesions	Slower than energy devices; operator-dependent
In-bag contained power morcellation [18]	Encloses fibroid in a containment bag before morcellation, preventing fragment spread	RCT (n=200): 0% parasitic myomas vs. 8% with uncontained (p<0.001)	Eliminates intra-abdominal seeding; addresses sarcoma risk	Adds 10-15 min; requires specialized bag and training
Double-layer myometrial closure [19]	First restores deep myometrium; second imbricates seromuscular layer to reinforce wall and minimize defects	Meta-analysis of 14 RCTs: adhesions 18% vs. 36% (p=0.01)	Stronger repair; halves adhesion formation	Slightly longer suture time
Laparoscopic vs. open myomectomy [20]	Comparison of minimally invasive to open approach in high-burden fibroid cases	Systematic reviews: -150 mL blood loss; -2.5 d hospital stay; 3% conversion; +30-45 min operative time	Less pain; faster recovery; better cosmetics	Longer operative time
Robotic-assisted myomectomy [21]	Uses wristed instruments and 3D optics for complex enucleation and suturing	Retrospective series: similar EBL; fewer conversions in dense uteri	Enhanced dexterity; stable 3D view	Higher cost; longer docking time; limited RCT evidence

Postoperative complications

Postoperative complications after extensive LM fall into several key areas, namely, haemorrhage leading to transfusion or conversion, infection, adhesion formation, uterine integrity, and morcellation-related sequelae, each of which demands vigilance and tailored prevention.

The blood transfusion rate in one of the largest cohorts of women undergoing LM for more than 10 fibroids (n>2,000) ranged from 6% to 15%, driven chiefly by preoperative anaemia, fibroid size or number, and intraoperative blood loss [22]. Most units were administered when haemoglobin fell below 8-9 g/dL or in symptomatic patients. Use of intraoperative cell salvage, restrictive transfusion triggers, and proactive correction of anaemia reduce this need. Conversion to open surgery occurred in approximately 3% of cases, typically for uncontrollable haemorrhage, extensive adhesions from prior surgery, or accidental injury to adjacent organs [22]. Early recognition of poor visualization or brisk bleeding and a low threshold for conversion can avert adverse outcomes.

A systematic review of 1,500 LM cases identified postoperative infection rates of 2-5%, predominantly urinary tract infections, surgical site infections, and, rarely, pelvic abscesses [23]. Perioperative prophylaxis, usually a first-generation cephalosporin administered within 60 minutes of incision, halves superficial wound infections. Meticulous sterilization of instruments, minimization of instrument exchanges, and irrigation with warmed saline further reduce contamination risk. In patients with morbid obesity or diabetes, extended-spectrum coverage and a 24-hour antibiotic course may be warranted [23].

Adhesions develop in up to 40% of women after myomectomy, compromising fertility and causing pelvic pain. In RCTs totalling 400 women, application of hyaluronic acid-based anti-adhesion gels immediately after uterine closure cut the incidence of de novo adhesions by about 30% compared with controls [24]. These agents act as a temporary barrier, allowing mesothelial healing. Surgeons should apply a thin, even film over all suture lines and limit the use of powdered gloves or talc-containing gauze. Second-look laparoscopy demonstrates that most residual adhesions are filmy and amenable to lysing.

Long-term strength of the uterine wall after LM is a paramount concern, particularly for women pursuing pregnancy. A meta-analysis of 600 observational cases determined a uterine rupture rate of 0.75%, virtually identical to open myomectomy cohorts [25]. Most ruptures occurred in the third trimester or at labour onset, often linked to deep or multiple-layer repairs. A robust two- or three-layer closure technique, avoidance of excessive electrocautery, and adherence to a minimum six-month interval before conception mitigate this risk. Patients should be counselled to plan caesarean delivery once fetal lung maturity is achieved [25].

Uncontained power morcellation carried an 8% rate of parasitic myoma implantation in a reported series [26]. In contrast, studies of in-bag contained morcellation report near-zero dissemination events. Surgeons deploy a containment bag, establish working space with insufflation, and morcellate within the sealed environment, adding only 10-15 minutes to the procedure. Vigilant retrieval of tissue fragments and copious irrigation after bag removal further guard against inadvertent seeding.

The percentage of hysterectomies during the LM of more than 10 fibroids is not directly reported in available literature; however, one study of 13,213 myomectomy procedures reported a 7.1% rate of concurrent hysterectomy and reported an increased risk of conversion in cases of multiple fibroids. We also experienced an increased risk of conversion to hysterectomy, considering the complexity of removing more than 10 uterine fibroids through a laparoscopic approach, for better safety and effectiveness of the surgical interventions [27].

By anticipating these complications and integrating evidence-based prevention measures, surgical teams can optimize safety and outcomes for women undergoing complex LM for more than 10 fibroids (Table 4).

**Table 4:** Overview of postoperative complications and management strategy

Postoperative complications and management strategy			
Postoperative complication	Haemorrhage → transfusion and conversion [22]	Cohort (n>2,000)	Transfusions 6-15%; conversions ~3%; correct anaemia; cell salvage
Postoperative complication	Infection [23]	Systematic review (n=1,500)	2-5%; use peri-op cephalosporin; sterile technique; irrigation
Postoperative complication	Adhesion formation [24]	RCTs (n=400)	Adhesions ↓ 30% with hyaluronic acid barrier; gentle handling; consider second-look
Postoperative complication	Uterine rupture [25]	Meta-analysis (n=600)	0.75%; multilayer closure; wait ≥6 months; plan caesarean
Postoperative complication	Parasitic myomas (morcellation) [26]	RCT (n=200)	8% (uncontained) vs. ~0% (contained); use in-bag technique

Fertility outcomes after myomectomy

Meta‐analysis data offer reassuring evidence that women retain strong reproductive potential after extensive LM. In the pooled analysis of 12 observational studies encompassing 850 women, the cumulative pregnancy rate within 12 months of surgery was 68% (95% CI: 63-73%), with a mean time to conception of seven months [28]. Importantly, only 0.5% of pregnancies were complicated by uterine rupture, typically occurring in the third trimester at old myomectomy sites, underscoring the safety of meticulous multilayer closure and appropriate inter‐pregnancy intervals [28].

The accidental endometrial breach during the LM of a uterine fibroid leads to increased incidence of placental accreta spectrum (PAS), placental malposition, preterm delivery, and placental abruption [29]. It is very important to recognize the endometrial breach early and repair of the same. The review article proposed strong consideration of elective caesarean section in case of multiple fibroids with potential endometrial breach for a safe fertility outcome [29].

A systematic review of 600 post‐LM pregnancies demonstrated a 60% live birth rate, statistically indistinguishable from cohorts undergoing open myomectomy [30]. Rates of spontaneous miscarriage (10-15%) and preterm delivery (8-12%) were likewise comparable, suggesting that minimally invasive access does not compromise endometrial receptivity or uterine distensibility [30].

Assisted reproductive technology (ART) further augments these outcomes in women whose fibroid burden or residual submucosal distortion might impede spontaneous conception. Cohort meta‐analyses of 450 women showed a 40% clinical pregnancy rate per in vitro fertilization (IVF) cycle after LM, surpassing expected rates in age‐matched women with untreated high‐volume fibroids, who typically achieve 25-30% per cycle [31]. Ovarian reserve and embryo quality remained unaffected by LM, provided adnexal structures were preserved.

Despite these encouraging figures, a Cochrane review of four small RCTs (total n=442) comparing laparoscopic, robotic, and open myomectomy could not definitively endorse one approach over another for optimizing fertility (Table 5). Variability in study designs, small sample sizes, and heterogeneous patient characteristics limited statistical power, and live birth was infrequently reported as a primary endpoint [32]. The authors called for larger, standardized trials with long‐term follow‐up focusing on time to pregnancy, obstetric complications, and patient‐centred metrics.

**Table 5:** Review of present evidence on fertility outcome after myomectomy LM: laparoscopic myomectomy; ART: assisted reproductive technique; IVF: in vitro fertilization; RCTs: randomized controlled trials

Outcome	Findings	Evidence (n)
Spontaneous pregnancy [28]	68% conceive within 12 months; mean time to conception 7 months; uterine rupture 0.5%	Pooled from 12 observational studies (n=850)
Live birth and obstetric outcomes [30]	60% live birth; spontaneous miscarriage 10-15%; preterm delivery 8-12%	Systematic review of post-LM pregnancies (n=600)
ART clinical pregnancy [31]	40% pregnancy rate per IVF cycle versus 25-30% expected baseline	Cohort meta-analysis of ART outcomes (n=450)
Surgical approach comparison [32]	No clear fertility advantage for laparoscopic vs. robotic vs. open myomectomy	Cochrane review of 4 RCTs (n=442)

The key takeaways for clinical practice include counselling of the patients that two‐thirds conceive within a year of LM, with live birth rates around 60%, regardless of fibroid count when surgery is expertly performed. It is advisable to delay the conception for six months post‐LM to allow uterine scar remodelling and vascular recovery; intervals shorter than four months have been linked to marginally higher rupture risk. The caesarean delivery should be planned for most patients with deep or multiple myometrial repairs to minimize labour stress on the scar.

Summary of review and key points

This review article focuses on multiple preoperative strategies, intraoperative haemostatic techniques, advanced surgical techniques, and fertility outcomes following LM of more than 10 uterine fibroids. Among the preoperative strategies, the authors recommend multimodal management including pharmacotherapy, interventional radiology, and optimization of haemoglobin to reduce blood loss as well as operative time. Among the intraoperative haemostatic techniques, various collaborative methods can be used to improve the precision in addition to reducing blood loss and operative time. This review article focuses on the importance of advanced surgical techniques to reduce thermal injury and adhesion formation as well as to support optimal healing which is beneficial for future pregnancy. The authors recommend detailed patient counselling and delaying the pregnancy for a safe healing period following LM and elective caesarean as the preferred mode of delivery.

## Conclusions

LM for the removal of 10 or more fibroids has evolved from an ambitious frontier procedure into a reproducible, safe, and fertility-preserving approach, provided it is anchored in high-quality evidence and executed by experienced teams. Multimodal preoperative optimization of individual patients, including medical treatment, targeted intraoperative haemostasis techniques, precise surgical techniques during LM, and vigilant postoperative management, improves fertility outcomes and overall patient safety. Adequate training with resource allocation is an important cost-effective approach to reduce morbidity and mortality. The authors recommend that quality-of-life assessments and patient-reported outcome measures should be integrated into future research to capture the broader impact on sexual, emotional, and reproductive health.
